# Bifurcation Analysis of a Delayed Infection Model with General Incidence Function

**DOI:** 10.1155/2019/1989651

**Published:** 2019-07-09

**Authors:** Suxia Zhang, Hongsen Dong, Jinhu Xu

**Affiliations:** School of Science, Xi'an University of Technology, Xi'an 710048, China

## Abstract

In this paper, an infection model with delay and general incidence function is formulated and analyzed. Theoretical results reveal that positive equilibrium may lose its stability, and Hopf bifurcation occurs when choosing delay as the bifurcation parameter. The direction of Hopf bifurcation and the stability of the periodic solutions are also discussed. Furthermore, to illustrate the numerous changes in the local stability and instability of the positive equilibrium, we conduct numerical simulations by using four different types of functional incidence, i.e., bilinear incidence, saturation incidence, Beddington–DeAngelis response, and Hattaf–Yousfi response. Rich dynamics of the model, such as Hopf bifurcations and chaotic solutions, are presented numerically.

## 1. Introduction

Mathematical modeling has been proven to be valuable in exploring mechanisms and dynamical behaviors of the viral infection process. The analysis of these models can provide insights into developing effective control strategies for infections and evaluating antiviral therapies. Based on the basic virus infection model introduced by Nowak et al. [1], various models have been formulated by many authors to describe the dynamics of virus population qualitatively and quantitatively, and lots of interesting phenomena have been discussed, such as the global stability of the equilibria, bifurcations, periodic oscillations, limit cycles, and the effects of time delays.

For models of virus infections, it is observed that the time delays introduced into them can predict the viral infection process better when compared to models without delays, such as the time between viral entry into a target cell and the production of subsequent viral particles, the time necessary for the newly produced virus to become mature and then infectious particles, and that needed to activate the immune response, all of which cannot be ignored when describing the interaction of them [2, 3]. In the field of virus dynamics, many models with discrete or distributed delays have been studied, showing that rich dynamics including stability switches, Hopf bifurcation, and chaotic oscillations can be generated by the mechanisms of time delays. Specifically, stability switches can occur due to a delayed immune response and exponentially decayed delay-dependent parameters [4–9], which leads to the switches in the stability of equilibria because of the change in the value of parameters related with these delays.

It is known that the function forms of the incidence rate of the infection have a crucial role in the modeling of the virus dynamics, which are important in determining qualitative behavior of the proposed models and in giving a reasonable description of the dynamics. Huang et al. [10] and Zhang and Xu [11] formulated a virus dynamical model with Beddington–DeAngelis (BD) infection rate *βxυ*/(1+*a*_1_ *x*+*a*_2_ *υ*), where *x* represents the concentration of uninfected cells and *υ* represents that of virus, with *a*_1_, *a*_2_ ≥ 0 being constants. This function is similar to the well-known Holling type II functional response, and the term *a*_2_ *υ* in the denominator reflects the mutual interference between viruses. In [12], Zhou and Cui used a Crowley–Martin (CM) function response of the form *βxυ*/(1+*a*_1_ *x*+*a*_2_ *υ*+*a*_1_ *a*_2_ *xυ*), with *a*_1_, *a*_2_, *a*_3_ ≥ 0, which are constants. It can be seen that in BD and CM functional responses, when *a*_1_ > 0, *a*_2_=0, these two functions are simplified to the Holling type II functional response. And when *a*_1_=0, *a*_2_ > 0, they express a saturation response. Moreover, when *a*_1_=0, *a*_2_=0, they are the mass action process (or Holling type I functional response). Other more general incidence rates have also been proposed. McCluskey and Yang used function *f*(*x*, *υ*) as incidence rate under some biologically motivated assumptions to study the global stability of a virus model [13]. Hattaf et al. in [14, 15] used an incidence rate *f*(*x*, *y*, *υ*) that covers a variety of incidence functions such as BD response when *a*_3_=0 and CM response when *a*_3_=*a*_1_ *a*_2_ if *f*(*x*, *y*, *υ*)=*βx*/(1+*a*_1_ *x*+*a*_2_ *υ*+*a*_3_ *xυ*), which will be called Hattaf–Yousfi response in the following, with *y* being infected cells.

In this paper, we will study the influence of general incidence function and time delay on the dynamical behaviors of an infection model. Motivated by the works of [7, 13], we will focus on the dynamics of a delayed model that incorporates the immune response in antiviral defense, time delay in activating the immune response for the virus, and two general incidence functions for the transmission of virus-to-cell and cell-to-cell, respectively. The main aim of this paper is to study stability switches of the positive equilibrium, which are generated by the mechanism of time delay, implying the existence of changes in local stability and the instability of this equilibrium. By using the characteristic equation with delay-dependent parameters, normal theory, and center manifold theorem, we can indicate the existence of pure imaginary roots and then Hopf bifurcation. Furthermore, to verify the theoretical results, numerical simulations are performed for four different forms of incidence functions, including bilinear incidence, saturation incidence, Beddington–DeAngelis response, and Hattaf–Yousfi functional response, respectively.

The organization of the paper is as follows. In the next section, we introduce the virus dynamic model. The boundedness and nonnegativity of solutions of the model as well as the existence and uniqueness of equilibria are discussed in Section 3. In Section 4, both the stabilities of two equilibria and the conditions for the existence of Hopf bifurcation are studied. Furthermore, numerical simulations are presented in Section 5. A brief summary is given in Section 6, and the properties of the Hopf bifurcation solutions have been provided in Appendix.

## 2. Model Formulation

To account for the possible effect of the latent period in viral infection, we introduce a constant time delay *τ* to represent the time needed to activate the immune response. In this model, two transmissions of virus-to-cell and cell-to-cell have been both taken into consideration, and we hope to employ the function of incidence rate given by more general form. Consequently, the recruitment of infected cells is given by two functions, i.e., *f*_1_(*u*, *v*)*v* and *f*_2_(*u*, *w*)*w*, which were used in [16–18], here *u*, *w*, and *v* denote the concentrations of uninfected cells, infected cells, and free virus particles. *z* is used to denote the concentration of CTL cells. Assume that the uninfected cells are produced at a constant rate *s*. *d*_1_, *d*_2_, *d*_3_, and *d*_4_ represent the death rates of uninfected cells, infected cells, free virus, and CTL cells. *p* denotes the killing rate of infected cells by CTL cells. *k* and *c* are the production rates of free viral particles and the effector cells, respectively. Then, we construct the following model:

(1) $$\begin{array}{c} \left\{\begin{array}{c} \frac{du}{dt}=s-f_{1}\left(u,v\right)v\left(t\right)-f_{2}\left(u,w\right)w\left(t\right)-d_{1}u\left(t\right),\\ \frac{dw}{dt}=f_{1}\left(u,v\right)v\left(t\right)+f_{2}\left(u,w\right)w\left(t\right)-d_{2}w\left(t\right)-pw\left(t\right)z\left(t\right),\\ \frac{dv}{dt}=kw\left(t\right)-d_{3}v\left(t\right),\\ \frac{dz}{dt}=cw\left(t-\tau \right)-d_{4}z\left(t\right), \end{array}\right. \end{array}$$

with initial conditions

(2) $$\begin{array}{c} u\left(\theta \right)=\varphi_{1}\left(\theta \right),\\ w\left(\theta \right)=\varphi_{2}\left(\theta \right),\\ v\left(\theta \right)=\varphi_{3}\left(\theta \right),\\ z\left(\theta \right)=\varphi_{4}\left(\theta \right),\\ \theta \in \left[-\tau ,0\right], \end{array}$$

where *φ*=(*φ*_1_, *φ*_2_, *φ*_3_, *φ*_4_) ∈ *C*([−*τ*, 0],  *ℝ*_+_^4^) is the Banach space of continuous functions mapping the interval [−*τ*, 0] into *ℝ*_+_^4^, with *φ*_*i*_(*θ*) ≥ 0 (*θ* ∈ [−*τ*, 0],  *i*=1,2,3,4), and all constant parameters are positive.

Function *f*_*i*_ : *ℝ*_+_^2^⟶*ℝ*_+_ (*i*=1,2) is continuously differentiable and is assumed to satisfy the following conditions:

(3) $$\begin{array}{c} f_{1}\left(0,v\right)=f_{2}\left(0,w\right)=0,\;\text{for all }v\geq 0\text{ and }w\geq 0, \end{array}$$

(4) $$\begin{array}{c} \frac{\partial f_{1}}{\partial u}>0,\frac{\partial f_{2}}{\partial u}>0,\text{and }\frac{\partial f_{1}}{\partial v}\leq 0,\frac{\partial f_{2}}{\partial w}\leq 0,\;\text{for all }u\geq 0,v\geq 0\text{ and }w\geq 0, \end{array}$$

(5) $$\begin{array}{c} \frac{\partial \left(f_{1}v\right)}{\partial v}=f_{1}+\frac{\partial f_{1}}{\partial v},\;v\geq 0, \end{array}$$

These fundamental assumptions are biologically motivated, and it is easy to check that class of functions *f*_1_ (and *f*_2_) satisfying these hypotheses include incidence functions such as *f*_1_=*βu* (mass incidence), *βu*/(1+*av*) (saturation incidence), *βu*/(1+*a*_1_ *u*+*a*_2_ *v*) (Beddington–DeAngelis response), and *βu*/(1+*a*_1_ *u*+*a*_2_ *v*+*a*_3_ *uv*) (Hattaf–Yousfi response).

## 3. Preliminaries and Equilibria

We denote the state space of (1) by *X*=*C*([−*τ*, 0], *ℝ*_+_^4^), equipped with the sup-norm *φ*=sup_−*τ*≤*θ*≤0_|*φ*(*θ*)|. For any *φ* ∈ *X*, the existence and uniqueness of the solution

(6) $$\begin{array}{c} Y\left(t,\varphi \right)=\left(u\left(t,\varphi \right),w\left(t,\varphi \right),v\left(t,\varphi \right),z\left(t,\varphi \right)\right), \end{array}$$

of model (1) with initial condition (2) follow from the standard theory of functional differential equations [19]. Furthermore, *X* is positively invariant for (1). In the following, we discuss the boundedness [20] and nonnegativity of solutions of model (1) as well as the existence of equilibria.

**Theorem 1 .**

The solutions *Y*(*t*, *φ*) of model (1) with initial condition (2) are nonnegative and ultimately uniformly bounded for all *t* ≥ 0.

**Proof**

For *u*(*t*), if there exists *t*_0_ such that *u*(*t*) > 0 for *t* ∈ [0, *t*_0_) and *u*(*t*_0_)=0, then it is obvious that *u*′(*t*_0_)=*s* > 0, which implies that for sufficiently small *ε* > 0, *u*(*t*) < 0 for *t* ∈ (*t*_0_ − *ε*, *t*_0_), contradicting with *u*(*t*) > 0 for all *t* ∈ [0, *t*_0_). Thus, it is valid that *u*(*t*) > 0 for all *t* ≥ 0. In order to show *w*(*t*) > 0, *v*(*t*) > 0, and *z*(*t*) > 0 for all *t* ≥ 0, assume that there exists *t*_1_ > 0 such that min{*w*(*t*_1_), *v*(*t*_1_), *z*(*t*_1_)} = 0 for the first time. Firstly, if *w*(*t*_1_)=0, then from the second equation of (1); we have

(7) $$\begin{array}{c} \frac{dw\left(t_{1}\right)}{dt}=f_{1}\left(u\left(t_{1}\right),v\left(t_{1}\right)\right),\;v\left(t_{1}\right)>0, \end{array}$$

and then there exists *ε*_1_ small enough such that (*dw*(*t*)/*dt*) > 0 for *t* ∈ (*t*_1_ − *ε*_1_, *t*_1_], which contradicts with the facts that *w*(*t*) > 0 for *t* ∈ (*t*_1_ − *ε*_1_, *t*_1_] and *w*(*t*_1_)=0. Thus *w*(*t*) > 0 for all *t* ≥ 0. For *v*(*t*) and *z*(*t*), since

(8) $$\begin{array}{c} v\left(t\right)=v\left(0\right)e^{-d_{3}t}+\int_{0}^{t}kw\left(\theta \right)e^{-d_{3}\left(t-\theta \right)}d\theta ,\\ z\left(t\right)=z\left(0\right)e^{-d_{4}t}+\int_{0}^{t}cw\left(\theta -\tau \right)e^{-d_{4}\left(t-\theta \right)}d\theta , \end{array}$$

it is obvious that *v*(*t*_1_)=0 or *z*(*t*_1_)=0 contradicts with the above expression about *v*(*t*) and *z*(*t*) at *t*=*t*_1_. Therefore, the solution *Y*(*t*, *φ*) with initial condition (2) is nonnegative for all *t* > 0.

To prove the boundedness of the solutions, letting *N*=*u*+*w*, we have

(9) $$\begin{array}{c} \frac{dN}{dt}=s-d_{1}u-d_{2}w-pwz\leq s-dN\;, \end{array}$$

where *d*=min{*d*_1_, *d*_2_}, then lim sup_*t*⟶*∞*_ *N* ≤ (*s*/*d*), implying that *u*(*t*) and *w*(*t*) are ultimately bounded. For *v*(*t*), it follows from the third equation of (1) that

(10) $$\begin{array}{c} \frac{dv}{dt}\leq \frac{ks}{d}-d_{3}v, \end{array}$$

which leads to lim sup_*t*⟶*∞*_ *v* ≤ (*ks*/*dd*_3_). Similarly, we have lim sup_*t*⟶*∞*_ *z* ≤ (*cs*/*dd*_4_). Therefore, the solutions of system (1) are ultimately uniformly bounded.

System (1) always has an infection-free equilibrium *E*_0_=(*u*_0_, 0,0,0), where *u*_0_=*s*/*d*_1_. In the following, we analyze the existence and uniqueness of the positive equilibrium *E* ^*∗*^=(*u* ^*∗*^, *w* ^*∗*^, *v* ^*∗*^, *z* ^*∗*^).

For convenience, we denote

(11) $$\begin{array}{c} f_{1}^{0}=f_{1}\left(u_{0},0\right),\\ f_{2}^{0}=f_{2}\left(u_{0},0\right),\\ f_{1}^{*}=f_{1}\left(u^{*},v^{*}\right),\\ f_{2}^{*}=f_{2}\left(u^{*},w^{*}\right). \end{array}$$

Define the basic reproductive number as

(12) $$\begin{array}{c} {ℛ}_{0}=\frac{kf_{1}^{0}}{d_{2}d_{3}}+\frac{f_{2}^{0}}{d_{2}}≔{ℛ}_{0}^{1}+{ℛ}_{0}^{2}. \end{array}$$

From biological viewpoint, *ℛ*_0_ can be divided into two parts, with *ℛ*_0_^1^ measuring the average number of secondary infected generation caused by an existing free virus and *ℛ*_0_^2^ being that caused by an infected cell, which give the basic reproduction number corresponding to virus-to-cell and cell-to-cell infections, respectively.

It is easy to obtain *v* ^*∗*^=(*k*/*d*_3_)*w* ^*∗*^ and *z* ^*∗*^=(*c*/*d*_4_)*w* ^*∗*^. By the first and second equations in (1), we have

(13) $$\begin{array}{c} s-d_{1}u^{*}-d_{2}w^{*}-pw^{*}z^{*}=0, \end{array}$$

which gives *u* ^*∗*^=(1/*d*_1_)[*s* − *w* ^*∗*^(*d*_2_+(*pc*/*d*_4_)*w* ^*∗*^)]. In order to get the infected steady state, it is necessary that *s* − *w* ^*∗*^(*d*_2_+(*pc*/*d*_4_)*w* ^*∗*^) ≥ 0, and then, we must have $0<w\leq \bar{w}$, with $\bar{w}=\left(-d_{2}d_{4}+d_{4}\sqrt{\Delta }\right)/2pc$ and Δ=*d*_2_^2^+(4*spc*/*d*_4_).

From the equation of *w*, it follows that

(14) $$\begin{array}{c} f_{1}^{*}v^{*}+f_{2}^{*}w^{*}-\left(d_{2}+\frac{pc}{d_{4}}w^{*}\right)w^{*}=0. \end{array}$$

Denote the left side of (11) as *G*(*w* ^*∗*^), and then the positive equilibrium of model (1) are given by *G*(*w* ^*∗*^)=0 for $w^{*}\in \left(0,\bar{w}\right]$. Noting that *G*(0)=0 and

(15) $$\begin{array}{c} G\left(\bar{w}\right)=-\left(d_{2}+\frac{pc}{d_{4}}\bar{w}\right),\;\bar{w}<0, \end{array}$$

for *u*=0 when $w=\bar{w}$, and it is sufficient to show that *G*′(0) > 0 if there is a positive equilibrium. In fact, when *ℛ*_0_ > 1, we have

(16) $$\begin{array}{c} G{}^{\prime }\left(0\right)=f_{1}\left(\frac{s}{d_{1}},0\right)\frac{k}{d_{3}}+f_{2}\left(\frac{s}{d_{1}},0\right)-d_{2}=d_{2}\left({ℛ}_{0}-1\right)>0, \end{array}$$

and then *G*(*w* ^*∗*^) is positive for sufficiently small *w* ^*∗*^; therefore, there exists an infected steady state *E* ^*∗*^.

Now we examine the derivative of *G*(*w* ^*∗*^) at *E* ^*∗*^. Noting that *f*_1_^*∗*^ *v* ^*∗*^+*f*_2_^*∗*^ *w* ^*∗*^=*d*_2_ *w* ^*∗*^+*pw* ^*∗*^ *z* ^*∗*^, and then

(17) $$\begin{array}{c} \frac{f_{1}^{*}v^{*}+f_{2}^{*}w^{*}}{w^{*}}=\frac{k\left(f_{1}^{*}v^{*}+f_{2}^{*}w^{*}\right)}{d_{3}v^{*}}=d_{2}+\frac{pc}{d_{4}}w^{*}, \end{array}$$

and thus, we have

(18) $$\begin{array}{c} G{}^{\prime }\left(w^{*}\right)=f_{1}^{*}\frac{k}{d_{3}}+\frac{\partial f_{1}^{*}}{\partial u}\left(-d_{2}-\frac{2pc}{d_{4}}w^{*}\right)\frac{k}{d_{3}}w^{*}+\frac{\partial f_{1}^{*}}{\partial v}\frac{k}{d_{3}}\frac{k}{d_{3}}w^{*}+f_{2}^{*}+\frac{\partial f_{2}^{*}}{\partial u}\left(-d_{2}-\frac{2pc}{d_{4}}w^{*}\right)w^{*}+\frac{\partial f_{2}^{*}}{\partial w}w^{*}-\left(d_{2}+\frac{2pc}{d_{4}}w^{*}\right)\\ =f_{1}^{*}\frac{k}{d_{3}}+f_{2}^{*}-\left(d_{2}+\frac{pc}{d_{4}}w^{*}\right)+T_{-}\\ =f_{1}^{*}\frac{k}{d_{3}}+f_{2}^{*}-f_{1}^{*}\frac{k}{d_{3}}-f_{2}^{*}\frac{kw^{*}}{d_{3}v^{*}}+T_{-}\\ =T_{-}, \end{array}$$

where

(19) $$\begin{array}{c} T_{-}=\frac{\partial f_{1}^{*}}{\partial u}\left(-d_{2}-\frac{2pc}{d_{4}}w^{*}\right)\frac{k}{d_{3}}w^{*}+\frac{\partial f_{1}^{*}}{\partial v}\frac{k}{d_{3}}\frac{k}{d_{3}}w^{*}+\frac{\partial f_{2}^{*}}{\partial u}\left(-d_{2}-\frac{2pc}{d_{4}}w^{*}\right)w^{*}+\frac{\partial f_{2}^{*}}{\partial w}w^{*}-\frac{pc}{d_{4}}w^{*}, \end{array}$$

denoting the sum of negative terms, and thus function *G* is strictly decreasing at each of its zeros. Suppose there exists more than one infected steady state, then there must exist an equilibrium ${\bar{E}}^{*}=\left({\bar{u}}^{*},{\bar{w}}^{*},{\bar{v}}^{*},{\bar{z}}^{*}\right)$ such that $G\left({\bar{w}}^{*}\right)\geq 0$, which contradicts with the above fact. Therefore, there is only one positive equilibrium *E* ^*∗*^ when the basic reproductive number *ℛ*_0_ > 1, and it is easy to verify that there exists no infection equilibrium when *ℛ*_0_ < 1. Then the following result is obtained.

**Theorem 2 .**

For system (1), when *ℛ*_0_ < 1, there exists only the infection-free equilibrium *E*_0_=((*s*/*d*_1_), 0,0,0). If *ℛ*_0_ > 1, there is a unique infection equilibrium *E* ^*∗*^=(*u* ^*∗*^, *w* ^*∗*^, *v* ^*∗*^, *z* ^*∗*^) as well as *E*_0_.

When *ℛ*_0_ > 1, by using the persistence theory in [21], it can be shown that system (1) is uniformly persistent.

## 4. Stability and Hopf Bifurcation

In this section, we will study the stability of the two equilibria and the conditions for the existence of Hopf bifurcation.

### 4.1. Stability of *E*_0_

Firstly, we linearize model (1) at the steady states to discuss its local stability. The Jacobian matrix leads to the following characteristic equation:

(20) $$\begin{array}{c} \left|\begin{array}{cccc} \lambda +d_{1}+\frac{\partial f_{1}}{\partial u}v+\frac{\partial f_{2}}{\partial u}w & f_{2}+\frac{\partial f_{2}}{\partial w}w & f_{1}+\frac{\partial f_{1}}{\partial v}v & 0\\ -\left(\frac{\partial f_{1}}{\partial u}v+\frac{\partial f_{2}}{\partial u}w\right) & \lambda +d_{2}+pz-f_{2}-\frac{\partial f_{2}}{\partial w}w & -\left(f_{1}+\frac{\partial f_{1}}{\partial v}v\right) & pw\\ 0 & -k & \lambda +d_{3} & 0\\ 0 & -ce^{-\lambda \tau } & 0 & \lambda +d_{4} \end{array}\right|=0, \end{array}$$

and thus, the linearization of system (1) at *E*_0_ can be expressed by

(21) $$\begin{array}{c} \left(\lambda +d_{1}\right)\left(\lambda +d_{4}\right)\left(\lambda^{2}+\left(d_{2}+d_{3}-f_{2}^{0}\right)\lambda +d_{2}d_{3}\left(1-{ℛ}_{0}\right)\right)=0. \end{array}$$

It is clear that (21) has two negative real roots *λ*_1_=−*d*_1_ and *λ*_2_=−*d*_4_. The stability of *E*_0_ is determined completely by using the following equation:

(22) $$\begin{array}{c} \lambda^{2}+\left(d_{2}+d_{3}-f_{2}^{0}\right)\lambda +d_{2}d_{3}\left(1-{ℛ}_{0}\right)=0. \end{array}$$

For the case *ℛ*_0_ < 1, the roots of (22) have only negative real parts. Then the infection-free equilibrium *E*_0_ is locally asymptotically stable. Otherwise, when *ℛ*_0_ > 1, equation (22) has at least one root with positive real part, which implies that *E*_0_ is unstable.

**Theorem 3 .**

If *ℛ*_0_ < 1, then the infection-free equilibrium *E*_0_ of model (1) is globally asymptotically stable, and it is unstable when *ℛ*_0_ > 1.

**Proof**

For a continuous and bounded function *f*(*t*), we define

(23) $$\begin{array}{c} f^{\infty }=\lim\underset{t⟶\infty }{\sup}f\left(t\right),\\ f_{\infty }=\lim\underset{t⟶\infty }{\inf}f\left(t\right). \end{array}$$

Then for any solution of (1), we have

(24) $$\begin{array}{c} 0\leq u_{\infty }\leq u^{\infty }<\infty ,\\ 0\leq w_{\infty }\leq w^{\infty }<\infty ,\\ 0\leq v_{\infty }\leq v^{\infty }<\infty ,\\ 0\leq z_{\infty }\leq z^{\infty }<\infty . \end{array}$$

By using the fluctuation lemma in [22], we know that there is a sequence {*t*_*n*_} with *t*_*n*_⟶*∞* such that

(25) $$\begin{array}{c} w\left(t_{n}\right)⟶w^{\infty },\\ \text{and }w{}^{\prime }\left(t_{n}\right)⟶0,\\ n⟶\infty . \end{array}$$

Substituting the sequence {*t*_*n*_} into the first equation of (1) and taking the limit gives

(26) $$\begin{array}{c} \underset{n⟶\infty }{\lim}u{}^{\prime }\left(t_{n}\right)\leq s-d_{1}\underset{n⟶\infty }{\lim}u\left(t_{n}\right), \end{array}$$

and then we have *u* ^*∞*^ ≤ (*λ*/*d*_1_)=*u* ^0^. A similar argument to the second and third equations of model (1) yields

(27) $$\begin{array}{c} d_{2}w^{\infty }\leq f_{1}\left(u^{\infty },v^{\infty }\right)v^{\infty }+f_{2}\left(u^{\infty },w^{\infty }\right)w^{\infty }, \end{array}$$

and

(28) $$\begin{array}{c} v^{\infty }=\frac{k}{d_{3}}w^{\infty }. \end{array}$$

By the assumption of (4), combining equality (28) into (27) gives

(29) $$\begin{array}{c} d_{2}w^{\infty }\leq f_{1}\left(u^{0},0\right)\frac{k}{d_{3}}w^{\infty }+f_{2}\left(u^{0},0\right)w^{\infty }, \end{array}$$

which leads to *d*_2_ *w* ^*∞*^ ≤ *d*_2_ *ℛ*_0_ *w* ^*∞*^. Noticing that *w* ^*∞*^ is nonnegative since it is the supremum of the function *w*(*t*), then there are possible cases of *w* ^*∞*^ > 0 or *w* ^*∞*^=0. If *w* ^*∞*^ > 0, then we have *ℛ*_0_ ≥ 1, which gives a contradiction. Therefore, *w* ^*∞*^=0 is valid. From (28), we have *v* ^*∞*^=0. When considering the fourth equation about *z*(*t*) in model (1), we obtain *z* ^*∞*^=0. As for *u*(*t*), we can find that lim_*t*⟶*∞*_ *u*(*t*)=*s*/*d*_1_ by applying the limit theory to the first equation in system (1), and thus, the proof is completed.

### 4.2. Stability of *E* ^*∗*^

For convenience, we introduce the following notations:

(30) $$\begin{array}{c} \frac{\partial f_{1}^{*}}{\partial u}v^{*}+\frac{\partial f_{2}^{*}}{\partial u}w^{*}=Q_{0},\\ \frac{\partial f_{1}^{*}}{\partial v}v^{*}+f_{1}^{*}=Q_{1},\\ \frac{\partial f_{2}^{*}}{\partial w}w^{*}+f_{2}^{*}=Q_{2}. \end{array}$$

From the assumption (5), it follows that *Q*_*i*_ ≥ 0 (*i*=0,1,2).

In the following calculation, we will use the following equilibrium equations:

(31) $$\begin{array}{c} \left\{\begin{array}{c} s-f_{1}^{*}v^{*}-f_{2}^{*}w^{*}-d_{1}u^{*}=0,\\ f_{1}^{*}v^{*}+f_{2}^{*}w^{*}=d_{2}w^{*}+pw^{*}z^{*},\\ kw^{*}=d_{3}v^{*},\\ cw^{*}=d_{4}z^{*}. \end{array}\right. \end{array}$$

Then, we have

(32) $$\begin{array}{c} d_{2}+pz^{*}-Q_{2}=d_{2}+pz^{*}-f_{2}^{*}-\frac{\partial f_{2}^{*}}{\partial w}w^{*}\\ =\frac{f_{1}^{*}v^{*}+f_{2}^{*}w^{*}}{w^{*}}-f_{2}^{*}-\frac{\partial f_{2}^{*}}{\partial w}w^{*}\\ =f_{1}^{*}\frac{k}{d_{3}}-\frac{\partial f_{2}^{*}}{\partial w},w^{*}>0,\\ -kf_{1}^{*}=-k\frac{d_{2}w^{*}+pw^{*}z^{*}-f_{2}^{*}w^{*}}{v^{*}}\\ =-k\left(d_{2}\frac{d_{3}}{k}+pz^{*}\frac{d_{3}}{k}-f_{2}^{*}\frac{d_{3}}{k}\right)\\ =-d_{2}d_{3}-d_{3}pz^{*}+d_{3}f_{2}^{*},\\ d_{3}f_{2}^{*}-d_{3}Q_{2}=-d_{3}\frac{\partial f_{2}^{*}}{\partial w},w^{*}>0. \end{array}$$

Linearized model (1) at the infection equilibrium *E* ^*∗*^ and the characteristic equation can be expressed as

(33) $$\begin{array}{c} \lambda^{4}+A_{3}\lambda^{3}+A_{2}\lambda^{2}+A_{1}\lambda +A_{0}+\left(B_{2}\lambda^{2}+B_{1}\lambda +B_{0}\right)e^{-\lambda \tau }=0, \end{array}$$

where *A*_*i*_=*A*_*i*_(*τ*) (*i*=0,1,2,3) and *B*_*j*_=*B*_*j*_(*τ*)(*j*=0,1,2) with

(34) $$\begin{array}{c} A_{3}=d_{1}+d_{3}+d_{4}+Q_{0}+d_{2}+pz^{*}-Q_{2},\\ A_{2}=\left(d_{1}+Q_{0}\right)\left(d_{3}+d_{4}+d_{2}+pz^{*}-Q_{2}\right)+d_{3}d_{4}+d_{4}\left(d_{2}+pz^{*}-Q_{2}\right)+Q_{0}Q_{2}-kv^{*}\frac{\partial f_{1}^{*}}{\partial v}-d_{3}w^{*}\frac{\partial f_{2}^{*}}{\partial w},\\ A_{1}=d_{3}d_{4}\left(d_{1}+Q_{0}\right)+Q_{0}Q_{2}\left(d_{3}+d_{4}\right)+d_{4}\left(d_{1}+Q_{0}\right)\left(d_{2}+pz^{*}-Q_{2}\right)+\left(d_{1}+d_{4}+Q_{0}\right)\left(-kv^{*}\frac{\partial f_{1}^{*}}{\partial v}-d_{3}w^{*}\frac{\partial f_{2}^{*}}{\partial w}\right),\\ A_{0}=d_{4}\left(d_{1}+Q_{0}\right)\left(-kv^{*}\frac{\partial f_{1}^{*}}{\partial v}-d_{3}w^{*}\frac{\partial f_{2}^{*}}{\partial w}\right)+d_{3}d_{4}Q_{0}Q_{2}+kQ_{1},\\ \text{and }B_{2}=pw^{*}c,\\ B_{1}=pw^{*}c\left(d_{1}+d_{3}+Q_{0}\right),\\ B_{0}=pw^{*}cd_{3}\left(d_{1}+Q_{0}\right). \end{array}$$

It can be seen that *A*_*i*_(*i*=0,1,2,3) and *B*_*j*_(*j*=0,1,2) are all positive. When the delay *τ*=0, *A*_*i*_(*i*=0,1,2,3) and *B*_*j*_(*j*=0,1,2) are independent with *τ* and the characteristic equation (33) can be reduced to

(35) $$\begin{array}{c} \lambda^{4}+A_{3}\lambda^{3}+\left(A_{2}+B_{2}\right)\lambda^{2}+\left(A_{1}+B_{1}\right)\lambda +A_{0}+B_{0}=0. \end{array}$$

By using the Routh–Hurwitz criterion, we know that all solutions of (35) have negative real parts if and only if the following conditions are satisfied:

(36) $$\begin{array}{c} H_{1}=A_{3}\left(A_{2}+B_{2}\right)-\left(A_{1}+B_{1}\right)>0,\\ H_{2}=A_{3}\left(A_{2}+B_{2}\right)\left(A_{1}+B_{1}\right)-A_{3}^{2}\left(A_{0}+B_{0}\right)-{\left(A_{1}+B_{1}\right)}^{2}>0. \end{array}$$

Let *f*=*f*_1_ *v*+*f*_2_ *w*,  *f* ^*∗*^=*f*_1_^*∗*^ *v* ^*∗*^+*f*_2_^*∗*^ *w* ^*∗*^,  *f*_*u*_=*f*(*u*, *w* ^*∗*^, *v* ^*∗*^) and assume

(37) $$\begin{array}{c} \left(1-\frac{f}{f_{u}}\right)\left(\frac{f}{f_{u}}-\frac{v}{v^{*}}\right)\geq 0,\;\text{for all }u,w,v>0. \end{array}$$

**Theorem 4 .**

If (37) holds and *τ*=0, then the infection equilibrium *E* ^*∗*^ is globally asymptotically stable when *ℛ*_0_ > 1.

**Proof**

Construct a Lyapunov functional as

(38) $$\begin{array}{c} L\left(t\right)=u-u^{*}-\int_{u^{*}}^{u}\frac{f^{*}}{f_{s}}ds+w-w^{*}\ln\frac{w}{w^{*}}+\frac{\left(d_{2}+pz^{*}\right)}{k}\left(v-v^{*}\ln\frac{v}{v^{*}}\right)+\frac{pz^{*}}{c}\left(z-z^{*}\ln\frac{z}{z^{*}}\right), \end{array}$$

and then calculating the time derivation of *L*(*t*) along system (1) leads to

(39) $$\begin{array}{c} \frac{dL\left(t\right)}{dt}=d_{1}u^{*}\left(1-\frac{f^{*}}{f_{u}}\right)\left(1-\frac{u}{u^{*}}\right)+f^{*}\left(\frac{f}{f_{u}}-\frac{v}{v^{*}}\right)+pz^{*}\left(-w\frac{z^{*}}{z}-w\frac{z}{z^{*}}\right)+2pwz^{*}+3f^{*}-f^{*}\frac{wv^{*}}{w^{*}v}-f^{*}\frac{f^{*}}{f_{u}}-f^{*}\frac{fw^{*}}{f^{*}w}\\ =d_{1}u^{*}\left(1-\frac{f^{*}}{f_{u}}\right)\left(1-\frac{u}{u^{*}}\right)-pwz^{*}\left(\frac{z^{*}}{z}+\frac{z}{z^{*}}-2\right)+f^{*}\left(g\left(\frac{f}{f_{u}}\right)-g\left(\frac{v}{v^{*}}\right)\right)-f^{*}\left(g\left(\frac{wv^{*}}{w^{*}v}\right)+g\left(\frac{f^{*}}{f_{u}}\right)+g\left(\frac{w^{*}f}{wf^{*}}\right)\right), \end{array}$$

with *g*(*x*)=*x* − 1 − ln *x*. By using (4), (37), and the property of function *g*(*x*), we have (1 − (*f* ^*∗*^/*f*_*u*_))(1 − (*u*/*u* ^*∗*^)) ≤ 0 and *g*(*f*/*f*_*u*_) ≤ *g*(*v*/*v* ^*∗*^), which implies that (*dL*(*t*)/*dt*) ≤ 0 and it can verified that {*E* ^*∗*^} is the largest compact invariant set where (*dL*(*t*)/*dt*)=0. Therefore, *E* ^*∗*^ is globally asymptotically stable by using LaSalle's invariance principle.

However, if the delay *τ* ≠ 0, it may destabilize the infected steady state and lead to Hopf bifurcation, which will be discussed in the following subsection.

### 4.3. Hopf Bifurcation

It is shown that all roots of (33) locate in the left side of the imaginary axis if *τ*=0 and (36) is satisfied, which leads to local stability of the infected equilibrium *E* ^*∗*^. When *τ* increases from 0 to variant positive delays, it is possible that the roots of (35) pass through the imaginary axis and enter the right side in the complex plane. Therefore, the stability switch may occur, and it is necessary to study the transcendental equation (33) when *λ*=*iω*, which is a critical value under small perturbation. In the following analysis, we will study the occurrence of any possible stability switching in this case.

Substituting *λ*=*iω* into (33) and separating the real and imaginary parts give

(40) $$\begin{array}{c} \left\{\begin{array}{c} -\omega^{4}+A_{2}\omega^{2}-A_{0}=\left(B_{0}-B_{2}\omega^{2}\right)\cos\;\omega \tau +B_{1}\omega \;\sin\;\omega \tau ,\\ -A_{3}\omega^{3}+A_{1}\omega =\left(B_{0}-B_{2}\omega^{2}\right)\sin\;\omega \tau -B_{1}\omega \;\cos\;\omega \tau . \end{array}\right. \end{array}$$

Squaring and adding the two equalities lead to

(41) $$\begin{array}{c} F\left(\omega \right)=\omega^{8}+M_{3}\omega^{6}+M_{2}\omega^{4}+M_{1}\omega^{2}+M_{0}=0, \end{array}$$

where

(42) $$\begin{array}{c} M_{3}=A_{3}^{2}-2A_{2},\\ M_{2}=A_{2}^{2}+2A_{0}-2A_{1}A_{3}-B_{2}^{2},\\ M_{1}=A_{1}^{2}-2A_{2}A_{0}+2B_{2}B_{0}-B_{1}^{2},\\ M_{0}=A_{0}^{2}-B_{0}^{2}. \end{array}$$

Note that equation *F*(*ω*)=0 has at least one positive root when *M*_0_ < 0, i.e., *A*_0_ < *B*_0_. And let *z*=*ω* ^2^, then the characteristic equation (33) has a purely imaginary root *iω* which is equivalent to *F*(*z*)=0 has a positive real root *z*. Take the transformation *y*=*z*+(*M*_3_/4); then for *F*′(*z*)=0, it is equivalent to *y* ^3^+*m*_1_ *y*+*m*_0_=0 with *m*_1_=(*M*_2_/2) − (3*M*_3_^2^/16), *m*_0_=(*M*_3_^3^/32) − (*M*_3_ *M*_2_/8)+(*M*_1_/4), and it has roots of *y*_*i*_, correspondingly, *z*_*i*_=*y*_*i*_ − (*M*_3_/4) (*i*=1,2,3); thus we have the following result about the roots of *F*(*z*)=0 [23].

**Lemma 1 .**

Denoting Δ=(*m*_0_/2)^2^+(*m*_1_/3)^3^, for the roots of *F*(*z*)=0,

- If *M*_0_ < 0, then there exists at least one positive root
- If *M*_0_ ≥ 0 and Δ≥0, then there are positive roots if and only if *z*_1_ > 0 and *F*(*z*_1_) ≤ 0
- If *M*_0_ ≥ 0 and Δ<0, then there are positive roots if and only if there exists at least a positive *z*_*∗*_ ∈ {*z*_1_, *z*_2_, *z*_3_} such that *F*(*z*_*∗*_) ≤ 0

Now assume that *F*(*z*)=0 has four roots *z*_*i*_^*∗*^ (*i*=1,2,3,4) with *z*_*i*_^*∗*^ > 0; then by the relation of *z*=*ω* ^2^, we have the fact of $\omega_{i}=\sqrt{z_{i}^{*}}$. From the equalities in (40), we can express cos *ωτ* and sin *ωτ* as

(43) $$\begin{array}{c} \left\{\begin{array}{c} \cos\;\omega \tau =\frac{\left(\omega^{4}-A_{2}\omega^{2}+A_{0}\right)\left(B_{2}\omega^{2}-B_{0}\right)+B_{1}\omega \left(A_{3}\omega^{3}-A_{1}\omega \right)}{B_{1}^{2}\omega^{2}+{\left(B_{0}-B_{2}\omega^{2}\right)}^{2}}≜F_{c},\\ \sin\;\omega \tau =\frac{B_{1}\omega \left(-\omega^{4}+A_{2}\omega^{2}-A_{0}\right)+\left(B_{0}-B_{2}\omega^{2}\right)\left(-A_{3}\omega^{3}+A_{1}\omega \right)}{B_{1}^{2}\omega^{2}+{\left(B_{0}-B_{2}\omega^{2}\right)}^{2}}≜F_{s}. \end{array}\right. \end{array}$$

Then for *j*=0,1,2,3,…, we can obtain the following expression of delay *τ*:

(44) $$\begin{array}{c} \tau_{j}^{i}=\left\{\begin{array}{cc} \frac{\arccos\left(F_{c}\right)+2\pi j}{\omega_{i}}, & F_{s}\geq 0,\\ \frac{2\pi -\arccos\left(F_{c}\right)+2\pi j}{\omega_{i}},\; & F_{s}<0. \end{array}\right. \end{array}$$

**Lemma 2 .**

For the roots of characteristic equation (35), suppose conditions in (36) hold, then there are two possibilities:

- Equation (35) has only roots with negative real parts for $\tau \in \left[0,\underset{i,j}{\min}\left\{\tau_{j}^{i}\right\}\right)$ if any one of the following conditions (*c*_1_)∼(*c*_3_) holds
- Equation (35) has only roots with negative real parts for *τ* ≥ 0 if the conditions (*c*_1_)∼(*c*_3_) are not satisfied, with
    (*c*_1_) *M*_0_ < 0
    (*c*_2_) *M*_0_ ≥ 0, Δ ≥ 0, *z*_1_ > 0 and *F*(*z*_1_) < 0
    (*c*_3_) *M*_0_ ≥ 0, Δ < 0, there is a positive *z*_*∗*_ ∈ {*z*_1_, *z*_2_, *z*_3_} such that *F*(*z*_*∗*_) ≤ 0

From this lemma, we can see that the infection equilibrium is asymptotically stable for all *τ* ≥ 0 if the conditions (*c*_1_)∼(*c*_3_) are not satisfied. Otherwise, if one of these three conditions is satisfied, then the infection equilibrium is asymptotically stable for $\tau \in \left[0,\underset{i,j}{\min}\left\{\tau_{j}^{i}\right\}\right)$, and a Hopf bifurcation can occur at this equilibrium when *τ*=*τ* ^*∗*^ with *τ* ^*∗*^ being the critical value of *τ*_*j*_^*i*^.

**Theorem 5 .**

Suppose (36) holds, when *τ* ^*∗*^=*τ*_*j*_^*i*^(correspondingly *ω* ^*∗*^=*ω*_*i*_ for some *i*=1,2,3,4), then the characteristic equation (35) admits a pair of simple conjugate pure imaginary roots *λ*=*iω* ^*∗*^ and *λ*=−*iω* ^*∗*^, which crosses the imaginary axis from left to right (from right to left) if *δ* < 0(>0), where

(45) δ=signdRe λdττ=τ∗=signF′ω∗2.

**Proof**

Differentiating the characteristic equation (33) with respect to delay *τ* and arranging it can give the expression of (*dλ*/*dτ*)^−1^ as

(46) $$\begin{array}{c} {\left(\frac{d\lambda }{d\tau }\right)}^{-1}=\frac{4\lambda^{3}+3A_{3}\lambda^{2}+2A_{2}\lambda +A_{1}}{-\lambda \left(\lambda^{4}+A_{3}\lambda^{3}+A_{2}\lambda^{2}+A_{1}\lambda +A_{0}\right)}+\frac{2B_{2}\lambda +B_{1}}{\lambda \left(B_{2}\lambda^{2}+B_{1}\lambda +B_{0}\right)}-\frac{\tau }{\lambda }. \end{array}$$

Meanwhile, noticing the fact

(47) signdRe λdττ=τ∗=signRedλdτ−1τ=τ∗,

and then some computation leads to

(48) signdRe λdττ=τ∗=signRe4λ3+3A3λ2+2A2λ+A1−λλ4+A3λ3+A2λ2+A1λ+A0τ=τ∗+Re2B2λ+B1λB2λ2+B1λ+B0τ=τ∗=sign4ω∗6+3M3ω∗4+2M2ω∗2+M1B2ω∗2−B02+B12ω∗2=signF′ω∗2B2ω∗2−B02+B12ω∗2=signF′ω∗2,

which gives the desired result.

When model (1) undergoes stability switch at *E* ^*∗*^ for *τ*=*τ* ^*∗*^, the conditions and theorem for the direction and stability of Hopf bifurcation can be discussed and proven by the normal theory and center manifold theorem in [24], which are presented in Appendix.

## 5. Numerical Simulation

In model (1), the incidence rates for transmission of virus-to-cell and cell-to-cell are taken as the general form. By choosing four specific types of functions, i.e., bilinear incidence, saturation incidence, Beddington–DeAngelis response, and Hattaf–Yousfi response, we conduct numerical simulations to investigate the complex dynamics that model (1) can have, when taking time delay *τ* as the bifurcation parameter. The values of parameters, *d*_3_=2.4, *d*_4_=1.618, *p*=0.812, and *k*=200, remain the same in the following different cases.

**Figure 1:**
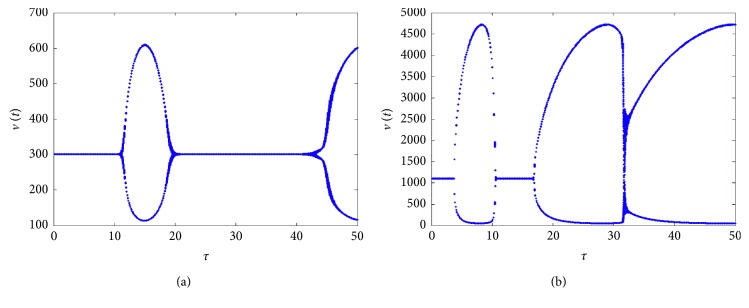
Bifurcation diagrams for model (1) with bilinear incidence rate. (a) *s*=2. (b) *s*=10. Other parameters are the same.

**Figure 2:**
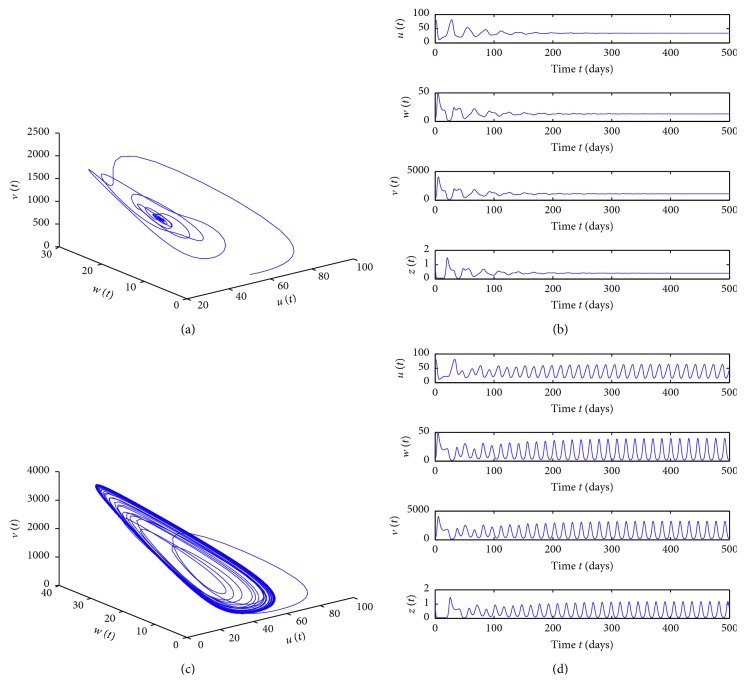
Solutions of the model for *τ*=15 (a, b) and *τ*=20 (c, d), corresponding to Figure 1(b).

**Figure 3:**
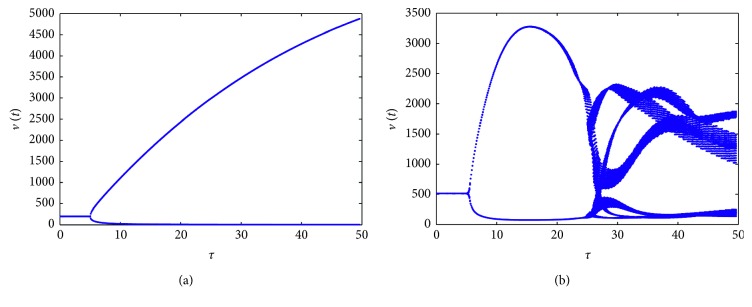
Bifurcation diagrams for model (1) with saturation incidence rate. (a) *s*=10, *β*_1_=0.002, *β*_2_=0.003, *a*=1, and *b*=1. (b) *s*=20, *β*_1_=0.02, *β*_2_=0.03, *a*=0.1, and *b*=0.4. Other parameters are the same.

**Figure 4:**
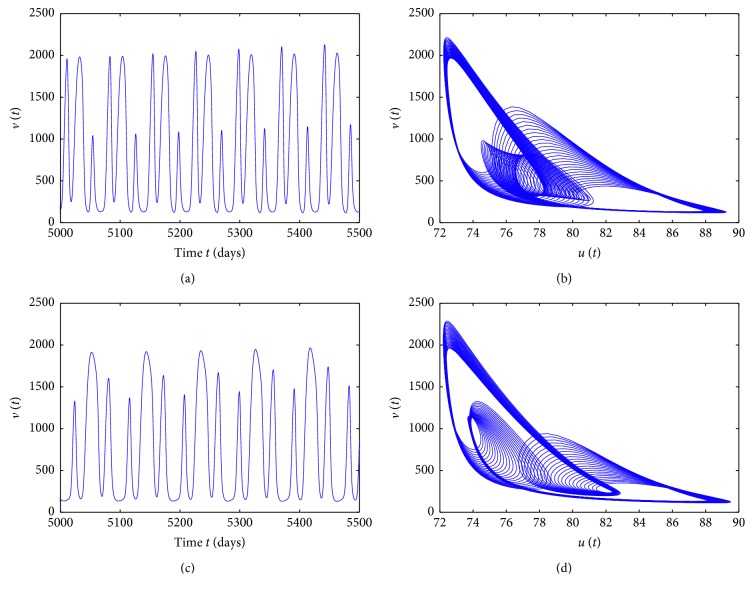
Solutions of the model for *τ*=35 (a, b) and *τ*=45 (c, d), corresponding to Figure 3(b).

**Figure 5:**
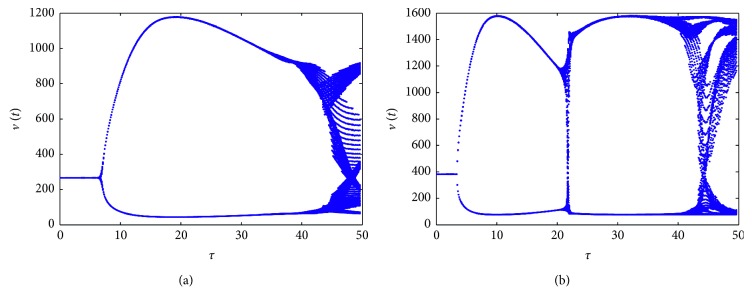
Bifurcation diagrams for model (1) with Beddington–DeAngelis response. (a) *a*_1_=0.08 and *a*_2_=0.5. (b) *a*_1_=0.01 and *a*_2_=0.2. Other parameters are the same.

**Figure 6:**
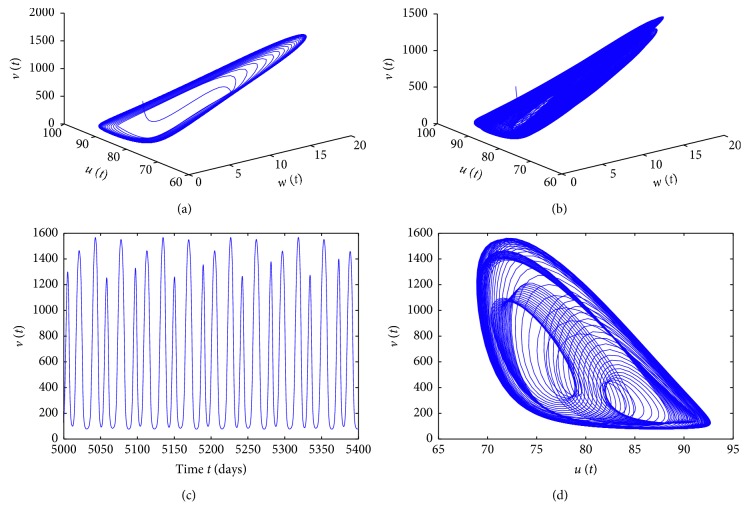
Solutions of the model for *τ*=10 (a), *τ*=22 (b), and *τ*=45 (c, d), corresponding to Figure 5(b).

**Figure 7:**
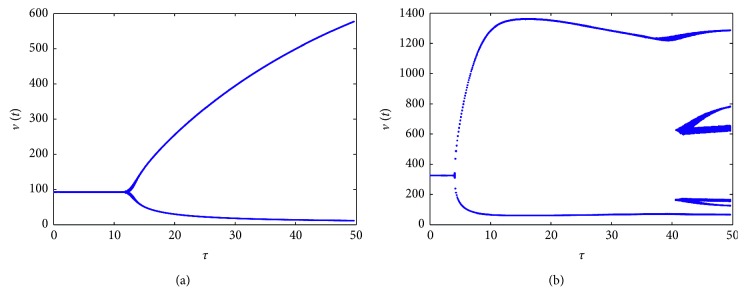
Bifurcation diagrams for model (1) with Hattaf–Yousfi response. (a) *s*=15, *c*=1, *d*_1_=0.01, *d*_2_=0.03, *β*_1_=0.5, *β*_2_=0.3, *a*_1_=1, *a*_2_=1, and *a*_3_=1. (b) *s*=20, *c*=1, *d*_1_=0.1, *d*_2_=0.3, *β*_1_=0.02, *β*_2_=0.03, *a*_1_=0.01, *a*_2_=0.3, and *a*_3_=0.001.

**Figure 8:**
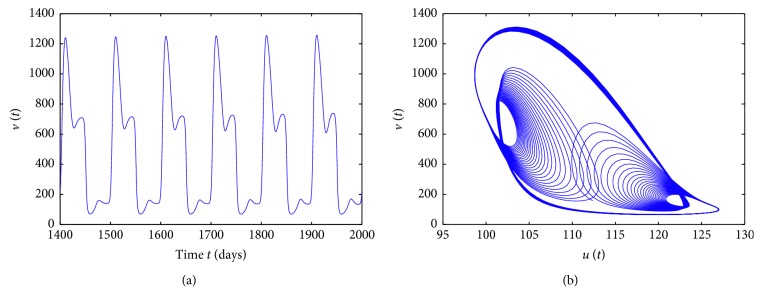
Solutions of the model for *τ* = 49, corresponding to Figure 7(b).

 
*Case 1*. Consider the functions of *f*_1_(*u*, *v*)=*β*_1_ *u* and *f*_2_(*u*, *w*)=*β*_2_ *u*, then the incidence rates have the bilinear forms of *β*_1_ *uv* and *β*_2_ *uw*. We take the other parameters as *c*=0.05, *d*_1_=0.01, *d*_2_=0.4, *β*_1_=0.00025, and *β*_2_=0.00065.  Figure 1 presents the different results for varying producing rate of uninfected cells, plotted with *s*=2 (Figure 1(a)) and *s*=10 (Figure 1(b)), which shows the effect of parameter *s* on dynamical behavior of the model in this case. In this figure, we can see that there exists periodic solutions bifurcated from the infection equilibrium. The maximal and minimal values of *v*(*t*) are denoted by the two curves, and the line implies the local stability of *E* ^*∗*^. It is clearly shown that the value of *v*(*t*) increases with that of *s*.  Figure 2 shows that the infection equilibrium *E* ^*∗*^ is asymptotically stable when *τ*=15 and a periodic solution exists when *τ*=20, which corresponds to the bifurcation diagram in Figure 1(b).
 
*Case 2*. The type of saturation incidence is used in this case, i.e., *f*_1_(*u*, *v*)=*β*_1_ *u*/(1+*av*) and *f*_2_(*u*, *w*)=*β*_2_ *u*/(1+*bw*); then the dynamical behaviors of the model are simulated in Figures 3 and 4, with the parameters taken as *c*=1, *d*_1_=0.01, and *d*_2_=0.03. In Figures 3(a), we set *s*=10, *β*_1_=0.002, *β*_2_=0.003, *a*=1, and *b*=1, and in Figure 3(b), we set *s*=20, *β*_1_=0.02, *β*_2_=0.03, *a*=0.1, and *b*=0.4. By the set of parameters, Figure 3(a) shows that the time delay does cause a bifurcation at *τ*=4.7403. The line is given by *v* ^*∗*^=198.0481, and the infected steady state *E* ^*∗*^ is locally asymptotically stable when *τ* < 4.7403. More complicated dynamics for model (1) are caused by the delay perturbation in Figure 3(b). A period-three solution occurs for *τ*=35 and *τ*=45, which are displayed clearly by time series of *v*(*t*) for the time interval [5000,5500] and their corresponding phase portraits of *v*(*t*) and *u*(*t*) plotted in Figure 4, respectively.
 
*Case 3*. Beddington–DeAngelis response is used in this case; here, *f*_1_(*u*, *v*)=*β*_1_ *u*/(1+*a*_1_ *u*+*a*_2_ *v*) and *f*_2_(*u*, *w*)=*β*_2_ *u*/(1+*a*_1_ *u*+*a*_2_ *w*). By this type of incidence function, chaotic motions also occur when increasing *τ* from 0 to 50 with *s*=20, *c*=1, *d*_1_=0.1, *d*_2_=0.3, *β*_1_=0.02, and *β*_2_=0.03. When altering parameter values of *a*_1_ and *a*_2_, it can lead to the different bifurcation diagrams, which can be seen in Figures 5 and 6. We take *a*_1_=0.08, *a*_2_=0.5 and *a*_1_=0.01, *a*_2_=0.2, respectively, and then the simulation results in Figure 5 show the effect of these two parameters on the system. From Figure 5, we can see that model (1) with these parameter values can experience complicated dynamics. Furthermore, for *τ*=10,22,  and 45 in Figure 5(b), the solution of the model is presented by its orbit, plot of time series, and phase portrait correspondingly in Figure 6. For *τ*=10, there exists a Hopf bifurcation, and when it increases to *τ*=22, chaotic motion occurs, shown in Figures 6(a) and 6(b). The plot of time series of *v*(*t*) and phase portrait of *v*(*t*) and *u*(*t*) are given to illustrate the dynamical behaviors when *τ*=45 in Figures 6(c) and 6(d), showing that there exists a period-five solution.
 
*Case 4*. When Hattaf–Yousfi response is used in this case, the functions take the form of *f*_1_(*u*, *v*)=*β*_1_ *u*/(1+*a*_1_ *u*+*a*_2_ *v*+*a*_3_ *uv*) and *f*_2_(*u*, *w*)=*β*_2_ *u*/(1+*a*_1_ *u*+*a*_2_ *w*+*a*_3_ *uw*), respectively. If the parameter values are chosen as *s*=15, *c*=1, *d*_1_=0.01, *d*_2_=0.03, *β*_1_=0.5, *β*_2_=0.3, *a*_1_=1, *a*_2_=1, and *a*_3_=1, only Hopf bifurcation exists and no chaotic motions occur when *τ* ∈ [0,50], shown in Figure 7(a). For a different set of values, let *s*=20, *c*=1, *d*_1_=0.1, *d*_2_=0.3, *β*_1_=0.02, *β*_2_=0.03, *a*_1_=0.01, *a*_2_=0.3, and *a*_3_=0.001, then the resulting bifurcation diagram in Figure 7(b) shows that system (1) can have solution of period three.  Figure 8 gives the time series of *v*(*t*) and phase portrait of *v*(*t*) and *u*(*t*) for *τ*=49 to illustrate the solution of system (1), which corresponds to Figure 7(b).

## 6. Discussion

To model the mechanisms of infectious diseases mathematically and explore dynamical behaviors of infection processes, some types of functional incidence have been used in [10, 12–15], which play an important role in determining qualitative behaviors of the proposed models and in giving reasonable descriptions of the dynamics. General incidence rate has been introduced into many epidemic models with an aim to include different situations as much as possible. In our model, we incorporate the effect of time delay needed to activate the immune response for the virus and two general incidence functions for the transmission of virus-to-cell and cell-to-cell. The basic reproductive number, which works as a crucial threshold and determines the dynamics of the model, has been defined as the sum of two parts related with infections of virus-to-cell and cell-to-cell, respectively.

In this paper, we are concerned with the stabilities of two equilibria and the existence of Hopf bifurcation through which the positive equilibrium loses its stability and periodic solutions occur. In the analysis, the time delay is chosen as the bifurcation parameter, which can destabilize the positive equilibrium when it increases. By using the characteristic equation with delay-dependent parameters, normal theory, and center manifold theorem, the existence of pure imaginary roots is verified and then the system experiences Hopf bifurcation. To illustrate the dynamical behavior of stability switches, simulations are conducted to show the process numerically when taking general incidence function as four specific types of bilinear incidence, saturation incidence, Beddington–DeAngelis response, and Hattaf–Yousfi response. In each case, the dynamical behavior is simulated when increasing *τ* from 0 to 50, and two sets of parameters are taken to compare the bifurcation results. Furthermore, for some fixed values of *τ*, we also present the plot of time series, phase portrait, or solution orbit to show the complicated dynamics. The effect of delay perturbation and different forms of incidence can be seen in the figures.

It should be noted that when other factors are taken into consideration for more realistic mechanism, such as the delays describing the intracellular latency for virus-to-cell infection and cell-to-cell infection, or varying producing rate of the uninfected cells instead of constant, the model can be extended reasonably and the dynamical analysis will become more challenging than the present one, which we may discuss in future work.

## Data Availability

No data were used to support the article.

## Appendix

Theorem 6 . For system (1),

- The direction of Hopf bifurcation is determined by the sign of *μ*_2_, i.e., it is a supercritical bifurcation when *μ*_2_ > 0 and a subcritical bifurcation when *μ*_2_ < 0.
- The stability of the bifurcated periodic solution is determined by ${\bar{\beta }}_{2}$, i.e., the periodic solution is stable when ${\bar{\beta }}_{2}<0$ and unstable when ${\bar{\beta }}_{2}>0$.
- The periodic of bifurcated periodic solutions is determined by *T*_2_, i.e., the period increases when *T*_2_ > 0 and decreases when *T*_2_ < 0, with

(A.1) c10=i2ωτ∗g20g11−2g112−g0223+g212,μ2=−Rec10Reλ′τ∗,β¯2=2Rec10,T2=Imc10+μ2Imλ′τ∗ωτ∗.

Proof In order to rewrite model (1) as a functional differential equation in *C*=*C*([−1,0], *ℝ* ^4^), we take the following transformations for variables and time scale:

(A.2) $$\begin{array}{c} x_{1}\left(t\right)=u\left(\tau t\right)-u^{*},\\ x_{2}\left(t\right)=w\left(\tau t\right)-w^{*},\\ x_{3}\left(t\right)=v\left(\tau t\right)-v^{*},\\ x_{4}\left(t\right)=z\left(\tau t\right)-z^{*},\\ \tau =\tau^{*}+\mu . \end{array}$$

Then model (1) becomes the following form:

(A.3) $$\begin{array}{c} \frac{dx}{dt}=L_{\mu }\left(x_{t}\right)+f\left(\mu ,x_{t}\right), \end{array}$$

where *x*(*t*)=(*x*_1_(*t*), *x*_2_(*t*), *x*_3_(*t*), *x*_4_(*t*))^T^ ∈ *ℝ* ^4^, *L*_*μ*_ : *C*⟶*ℝ* ^4^, *f* : *ℝ* × *C*⟶*ℝ* ^4^,

(A.4) $$\begin{array}{c} L_{\mu }\left(\phi \right)=\left(\tau^{*}+\mu \right)B_{1}\phi \left(0\right)+\left(\tau^{*}+\mu \right)B_{2}\phi \left(-1\right), \end{array}$$

with

(A.5) $$\begin{array}{c} B_{1}=\left(\begin{array}{cccc} -\left(d_{1}+Q_{0}\right) & -Q_{2} & -Q_{1} & 0\\ Q_{0} & -\left(d_{2}+pz^{*}-Q_{2}\right) & Q_{1} & -pw^{*}\\ 0 & k & -d_{3} & 0\\ 0 & 0 & 0 & -d_{4} \end{array}\right),\\ B_{2}=\left(\begin{array}{cccc} 0 & 0 & 0 & 0\\ 0 & 0 & 0 & 0\\ 0 & 0 & 0 & 0\\ 0 & c & 0 & 0 \end{array}\right), \end{array}$$

and

(A.6) $$\begin{array}{c} f\left(\mu ,\phi \right)=\left(\tau^{*}+\mu \right)\left(\begin{array}{c} -h\left(\phi \left(0\right)\right)\\ h\left(\phi \left(0\right)\right)-p\phi_{2}\left(0\right)\phi_{4}\left(0\right)\\ 0\\ 0 \end{array}\right), \end{array}$$

where *ϕ*(*θ*)=(*ϕ*_1_(*θ*), *ϕ*_2_(*θ*), *ϕ*_3_(*θ*), *ϕ*_4_(*θ*))^T^ ∈ *C* and

(A.7) $$\begin{array}{c} h\left(\phi \right)=\frac{1}{2}\left(\frac{\partial^{2}f_{1}}{\partial u^{2}}v^{*}+\frac{\partial^{2}f_{2}}{\partial u^{2}}w^{*}\right)\phi_{1}^{2}+\frac{1}{2}\left(\frac{\partial^{2}f_{2}}{\partial w^{2}}w^{*}+2\frac{\partial f_{2}}{\partial w}\right)\phi_{2}^{2}+\frac{1}{2}\left(\frac{\partial^{2}f_{1}}{\partial v^{2}}v^{*}+2\frac{\partial f_{1}}{\partial v}\right)\phi_{3}^{2}+\left(\frac{\partial f_{2}}{\partial u}+\frac{\partial^{2}f_{2}}{\partial u\;\partial w}w^{*}\right)\phi_{1}\phi_{2}+\left(\frac{\partial f_{1}}{\partial u}+\frac{\partial^{2}f_{1}}{\partial u\;\partial v}v^{*}\right)\phi_{1}\phi_{3}. \end{array}$$

By Riesz representation theorem, there exists a matrix components *η*(*θ*, *μ*) in *θ* ∈ [−1,0], which is a bounded variation function such that *L*_*μ*_ *ϕ*=∫_−1_^0^ *dη*(*θ*, *μ*)*ϕ*(*θ*) for *ϕ* ∈ *C*. As for (A.3), *η*(*θ*, *μ*) has the expression of

(A.8) $$\begin{array}{c} \eta \left(\theta ,\mu \right)=\left(\tau^{*}+\mu \right)B_{1}\delta \left(\theta \right)-\left(\tau^{*}+\mu \right)B_{2}\delta \left(\theta +1\right), \end{array}$$

where *δ* denotes the Dirac delta function. For *ϕ* ∈ *C* ^1^([−1,0], *ℝ* ^4^), we define

(A.9) $$\begin{array}{c} A\left(\mu \right)\phi =\left\{\begin{array}{cc} \frac{d\phi \left(\theta \right)}{d\theta }, & \theta \in \left[-1,0\right),\\ \int_{-1}^{0}d\eta \left(\mu ,s\right)\phi \left(s\right), & \theta =0, \end{array}\right. \end{array}$$

and

(A.10) $$\begin{array}{c} R\left(\mu \right)\phi =\left\{\begin{array}{cc} 0, & \theta \in \left[-1,0\right),\\ f\left(\mu ,\phi \right), & \theta =0. \end{array}\right. \end{array}$$

Then, model (1) is equivalent to

(A.11) $$\begin{array}{c} x_{t}^{\prime }=A\left(\mu \right)x_{t}+R\left(\mu \right)x_{t},x_{t}=x\left(t+\theta \right),\;\theta \in \left[-1,0\right]. \end{array}$$

Furthermore, for *ψ* ∈ *C* ^1^([0,1], (*ℝ* ^4^)^*∗*^), define

(A.12) $$\begin{array}{c} A^{*}\psi \left(s\right)=\left\{\begin{array}{cc} -\frac{d\psi \left(s\right)}{ds}, & s\in \left(0,1\right],\\ \int_{-1}^{0}d\eta^{\text{T}}\left(s,0\right)\psi \left(-s\right), & s=0, \end{array}\right. \end{array}$$

and a bilinear inner product

(A.13) $$\begin{array}{c} \langle \psi ,\phi \rangle =\bar{\psi }\left(0\right)\phi \left(0\right)-\int_{-1}^{0}\int_{\xi =0}^{\theta }\bar{\psi }\left(\xi -\theta \right)d\eta \left(\theta \right)\phi \left(\xi \right)d\xi , \end{array}$$

where *η*(*θ*)=*η*(*θ*, 0). *A*(0) and *A* ^*∗*^ are adjoint operators with eigenvalues ±*iωτ* ^*∗*^, and then we discuss the eigenvector of *A*(0) and *A* ^*∗*^ corresponding to these two eigenvalues, respectively. First, for *A*(0), if *q*(*θ*)=(1, *q*_1_, *q*_2_, *q*_3_)^T^ *e* ^*iθωτ*^*∗*^^ is the eigenvector corresponding to *iωτ* ^*∗*^, then it leads to *A*(0)*q*(*θ*)=*iωτ* ^*∗*^ *q*(*θ*), i.e.,

(A.14) $$\begin{array}{c} \tau^{*}\left(\begin{array}{cccc} i\omega +\left(d_{1}+Q_{0}\right) & Q_{2} & Q_{1} & 0\\ -Q_{0} & i\omega +\left(d_{2}+pz^{*}-Q_{2}\right) & -Q_{1} & pw^{*}\\ 0 & -k & i\omega +d_{3} & 0\\ 0 & -ce^{-i\omega \tau^{*}} & 0 & i\omega +d_{4} \end{array}\right)\left(\begin{array}{c} 1\\ q_{1}\\ q_{2}\\ q_{3} \end{array}\right)=\left(\begin{array}{c} 0\\ 0\\ 0\\ 0 \end{array}\right). \end{array}$$

Denoting the above equation as *τ* ^*∗*^ *A*_4×4_ *q*(*θ*)=0 and *a*_*ij*_ (*i*, *j*=1,2,3,4) being the element of *A*_4×4_ in *i*th row and *j*th column, then we have

(A.15) $$\begin{array}{c} q_{1}=\frac{-a_{11}}{a_{12}-\left(a_{13}a_{32}/a_{33}\right)},\\ q_{2}=\frac{-a_{32}q_{1}}{a_{33}},\\ q_{3}=\frac{-a_{42}q_{1}}{a_{44}}. \end{array}$$

Similarly, for *A* ^*∗*^, if *q* ^*∗*^(*s*)=*D*(1, *q*_1_^*∗*^, *q*_2_^*∗*^, *q*_3_^*∗*^)*e* ^*isωτ*^*∗*^^ is the eigenvector of *A* ^*∗*^ corresponding to −*iωτ* ^*∗*^, we can obtain

(A.16) $$\begin{array}{c} q_{1}^{*}=\frac{-b_{11}}{b_{12}},\\ q_{2}^{*}=\frac{-b_{32}\left(q_{1}^{*}-1\right)}{b_{33}},\\ q_{3}^{*}=\frac{-b_{42}q_{1}^{*}}{b_{44}}, \end{array}$$

where *b*_*ij*_ (*i*, *j*=1,2,3,4) is the element of matrix *B* in *i*th row and *j*th column and

(A.17) $$\begin{array}{c} B=\left(\begin{array}{cccc} -i\omega +\left(d_{1}+Q_{0}\right) & -Q_{0} & 0 & 0\\ Q_{2} & -i\omega +\left(d_{2}+pz^{*}-Q_{2}\right) & -k & -ce^{i\omega \tau^{*}}\\ Q_{1} & -Q_{1} & -i\omega +d_{3} & 0\\ 0 & pw^{*} & 0 & -i\omega +d_{4} \end{array}\right). \end{array}$$

Since 〈*q* ^*∗*^(*s*), *q*(*θ*)〉=1, we can choose

(A.18) $$\begin{array}{c} D=\frac{1}{1+{\bar{q}}_{1}q_{1}^{*}+{\bar{q}}_{2}q_{2}^{*}+{\bar{q}}_{3}q_{3}^{*}+\tau^{*}cq_{1}{\bar{q}}_{3}^{*}e^{-i\omega \tau^{*}}}. \end{array}$$

In the following, by using the notations in [24], we compute the center manifold *C*_0_ when *μ*=0. Correspondingly, assuming *x*_*t*_(*θ*)=(*x*_1*t*_(*θ*), *x*_2*t*_(*θ*), *x*_3*t*_(*θ*), *x*_4*t*_(*θ*)) be the solution of (A.6), and define *z*(*t*) and *W*(*t*, *θ*) as

(A.19) zt=q∗,xt,Wt,θ=xtθ−2Reztqθ.

Then on the center manifold *C*_0_, we have

(A.20) $$\begin{array}{c} W\left(t,\theta \right)=W\left(z,\bar{z},\theta \right)=W_{20}\frac{z^{2}}{2}+W_{11}\left(\theta \right)z\bar{z}+W_{02}\left(\theta \right)\frac{{\bar{z}}^{2}}{2}+\cdots , \end{array}$$

and

(A.21) xtθ=Wt,θ+2Reztqθ=1,q1,q2,q3Teiωτ∗θz+1,q¯1,q¯2,q¯3Te−iωτ∗θz¯+W20z22+W11θzz¯+W02θz¯22+⋯,

with *z* and $\bar{z}$ being local coordinates for *C*_0_ in the direction *q* ^*∗*^ and ${\bar{q}}^{*}$. Thus,

(A.22) $$\begin{array}{c} x_{1t}\left(0\right)=z+\bar{z}+W_{20}^{\left(1\right)}\frac{z^{2}}{2}+W_{11}^{\left(1\right)}\left(\theta \right)z\bar{z}+W_{02}^{\left(1\right)}\left(\theta \right)\frac{{\bar{z}}^{2}}{2}+O\left({\left|\left(z,\bar{z}\right)\right|}^{3}\right),\\ x_{2t}\left(0\right)=q_{1}z+{\bar{q}}_{1}\bar{z}+W_{20}^{\left(2\right)}\frac{z^{2}}{2}+W_{11}^{\left(2\right)}\left(\theta \right)z\bar{z}+W_{02}^{\left(2\right)}\left(\theta \right)\frac{{\bar{z}}^{2}}{2}+O\left({\left|\left(z,\bar{z}\right)\right|}^{3}\right),\\ x_{3t}\left(0\right)=q_{2}z+{\bar{q}}_{2}\bar{z}+W_{20}^{\left(3\right)}\frac{z^{2}}{2}+W_{11}^{\left(3\right)}\left(\theta \right)z\bar{z}+W_{02}^{\left(3\right)}\left(\theta \right)\frac{{\bar{z}}^{2}}{2}+O\left({\left|\left(z,\bar{z}\right)\right|}^{3}\right),\\ x_{4t}\left(0\right)=q_{3}z+{\bar{q}}_{3}\bar{z}+W_{20}^{\left(4\right)}\frac{z^{2}}{2}+W_{11}^{\left(4\right)}\left(\theta \right)z\bar{z}+W_{02}^{\left(4\right)}\left(\theta \right)\frac{{\bar{z}}^{2}}{2}+O\left({\left|\left(z,\bar{z}\right)\right|}^{3}\right). \end{array}$$

When considering real solution *x*_*t*_ ∈ *C*_0_ of (A.6), we can obtain

(A.23) z′t=iωτ∗z+q¯∗θ,f0,Wz,z¯,θ+2Rezqθ=iωτ∗z+q¯∗0f0,Wz,z¯,0+2Rezq0 ≕ iωτ∗z+q¯∗0f0z,z¯.

Let

(A.24) $$\begin{array}{c} g\left(z,\bar{z}\right)={\bar{q}}^{*}\left(0\right)f_{0}\left(z,\bar{z}\right)=g_{20}\frac{z^{2}}{2}+g_{11}z\bar{z}+g_{02}\frac{{\bar{z}}^{2}}{2}+g_{21}\frac{z^{2}\bar{z}}{2}+\cdots . \end{array}$$

On the other side, note that $g\left(z,\bar{z}\right)={\bar{q}}^{*}\left(0\right)f_{0}\left(z,\bar{z}\right)={\bar{q}}^{*}\left(0\right)f_{0}\left(0,x_{t}\right)$. Then by using the expression of *f*(*μ*, *ϕ*) in (A.4), we have

(A.25) $$\begin{array}{c} g\left(z,\bar{z}\right)=\tau^{*}\bar{D}\left[\left({\bar{q}}_{1}^{*}-1\right)h-{\bar{q}}_{1}^{*}px_{2t}\left(0\right)x_{4t}\left(0\right)\right]. \end{array}$$

Substituting the expressions of *h*(*x*_*t*_) and *x*_*t*_(0)=(*x*_1*t*_(0), *x*_2*t*_(0), *x*_3*t*_(0), *x*_4*t*_(0)) into (A.12) and comparing the coefficients of *z* ^2^, $z\bar{z}$, ${\bar{z}}^{2}$, and $z^{2}\bar{z}$ lead to

(A.26) g20=τ∗D¯q¯1∗−1Uu+q12Uw+q22Uv+2q1Uuw+2q2Uuv−2q¯1∗pq1q3,g11=τ∗D¯q¯1∗−1Uu+q1q¯1Uw+q2q¯2Uv+2Req1Uuw+2Req2Uuv−2q1∗pReq1q¯3,g02=τ∗D¯q¯1∗−1Uu+q¯12Uw+q¯22Uv+2q¯1Uuw+2q¯2Uuv−2q¯1∗pq¯1q¯3,g21=τ∗D¯q¯1∗−1UuW2010+2W1110+Uwq¯1W2020+2q1W1120+Uvq¯2W2030+2q2W1130+Uuw2W1120+q¯1W2010+W2020+2q1W1110+Uuv2W1130+q¯2W2010+W2030+2q2W1110−q1∗p2q1W1140+q¯3W2020+q¯1W2040+2q3W1120,

where

(A.27) $$\begin{array}{c} U_{u}=\frac{\partial^{2}f_{1}}{\partial u^{2}}v^{*}+\frac{\partial^{2}f_{2}}{\partial u^{2}}w^{*},\\ U_{w}=\frac{\partial^{2}f_{2}}{\partial w^{2}}w^{*}+2\frac{\partial f_{2}}{\partial w},\\ U_{v}=\frac{\partial^{2}f_{1}}{\partial v^{2}}v^{*}+2\frac{\partial f_{1}}{\partial v},\\ U_{uw}=\frac{\partial f_{2}}{\partial u}+\frac{\partial^{2}f_{2}}{\partial u\;\partial w}w^{*},\\ U_{uv}=\frac{\partial f_{1}}{\partial u}+\frac{\partial^{2}f_{1}}{\partial u\;\partial v}v^{*}. \end{array}$$

Next, in order to determine the value of *g*_21_, we compute *W*_20_ and *W*_11_ briefly. According to the results in [24], we have

(A.28) $$\begin{array}{c} W_{20}\left(\theta \right)=\frac{ig_{20}}{\omega \tau^{*}}q\left(0\right)e^{i\omega \tau^{*}\theta }+\frac{i{\bar{g}}_{02}}{3\omega \tau^{*}}\bar{q}\left(0\right)e^{-i\omega \tau^{*}\theta }+E_{1}e^{2i\omega \tau^{*}\theta },\\ W_{11}\left(\theta \right)=\frac{-ig_{11}}{\omega \tau^{*}}q\left(0\right)e^{i\omega \tau^{*}\theta }+\frac{i{\bar{g}}_{11}}{\omega \tau^{*}}\bar{q}\left(0\right)e^{-i\omega \tau^{*}\theta }+E_{2}, \end{array}$$

where *E*_*i*_=(*E*_*i*_^(1)^, *E*_*i*_^(2)^, *E*_*i*_^(3)^, *E*_*i*_^(4)^) (*i*=1,2) ∈ *ℝ*_4_ is a constant vector. Furthermore, it can be deduced that *E*_*i*_ satisfies

(A.29) $$\begin{array}{c} ME_{1}=2C,\\ \tilde{M}E_{1}=2\tilde{C}, \end{array}$$

where

(A.30) M=2iωI−B1−e−2iωτ∗B2,M˜=−B1−B2,C=−U,U−pq1q3,0,0T,C˜=−U˜,U˜−pReq1q¯3,0,0T,

with

(A.31) U=12Uu+12q12Uw+12q22Uv+q1Uuw+q2Uuv,U˜=12Uu+12q1q¯1Uw+12q2q¯2Uv+Req1Uuw+Req2Uuv.

By using Cramer's rule, we have

(A.32) $$\begin{array}{c} E_{1}^{\left(i\right)}=\frac{2\det\left(M_{i}\right)}{\det\left(M\right)},\\ E_{2}^{\left(i\right)}=\frac{2\det\left({\tilde{M}}_{i}\right)}{\det\left(\tilde{M}\right)}, \end{array}$$

where $M_{i}\left({\tilde{M}}_{i}\right)$ is the matrix that the *i*th column in $M\left(\tilde{M}\right)$ is substituted by $C\left(\tilde{C}\right)$. Thus *W*_20_(*θ*), *W*_11_(*θ*), and then *g*_21_ can be determined. Furthermore, the values of *c*_1_(0), *μ*_2_, ${\bar{\beta }}_{2}$, and *T*_2_ can be computed by using (A.1).
